# Maf-dependent bacterial flagellin glycosylation occurs before chaperone binding and flagellar T3SS export

**DOI:** 10.1111/mmi.12549

**Published:** 2014-03-04

**Authors:** Jennifer L Parker, Rebecca C Lowry, Narciso A S Couto, Phillip C Wright, Graham P Stafford, Jonathan G Shaw

**Affiliations:** 1 Department of Infection and Immunity, University of SheffieldSheffield, S10 2RX, UK; 2 Chemical and Biological Engineering, University of SheffieldSheffield, S1 3JD, UK; 3 School of Clinical Dentistry, University of SheffieldSheffield, S10 2EA, UK

## Abstract

Bacterial swimming is mediated by rotation of a filament that is assembled via polymerization of flagellin monomers after secretion via a dedicated flagellar Type III secretion system. Several bacteria decorate their flagellin with sialic acid related sugars that is essential for motility. *Aeromonas caviae* is a model organism for this process as it contains a genetically simple glycosylation system and decorates its flagellin with pseudaminic acid (Pse). The link between flagellin glycosylation and export has yet to be fully determined. We examined the role of glycosylation in the export and assembly process in a strain lacking Maf1, a protein involved in the transfer of Pse onto flagellin at the later stages of the glycosylation pathway. Immunoblotting, established that glycosylation is not required for flagellin export but is essential for filament assembly since non-glycosylated flagellin is still secreted. Maf1 interacts directly with its flagellin substrate *in vivo*, even in the absence of pseudaminic acid. Flagellin glycosylation in a flagellin chaperone mutant (*flaJ*) indicated that glycosylation occurs in the cytoplasm before chaperone binding and protein secretion. Preferential chaperone binding to glycosylated flagellin revealed its crucial role, indicating that this system has evolved to favour secretion of the polymerization competent glycosylated form.

## Introduction

Many bacterial species are motile through the action of flagella, a complex nanomachine in which a 10–15 μm helical filament extends from the cell surface and is anchored to a rotating basal body spanning the bacterial cell envelope that is powered by an ion gradient. The filament is composed of repeating subunits known as flagellins which are exported via a dedicated Type III secretion system (T3SS) (Erhardt *et al*., 2010). Bacterial flagellins have conserved N and C-terminal D0 and D1 domains that are required for polymerization, chaperone binding and export (Auvray *et al*., 2001). Secretion of flagellins and other T3SS substrates is controlled by the N-terminal export signal of the T3SS substrate and the presence of specific chaperones. The structural characterization of the N-terminal secretion signal is at present poorly understood (Arnold *et al*., 2009). The flagellin-specific chaperone FliS binds to the C-terminal helical domain of flagellin, stabilizing flagellins in the cytoplasm prior to export (Auvray *et al*., 2001). The importance of a fully functional flagellum and motility in the adherence of pathogenic bacteria to eukaryotic cells and colonization of the host, has been demonstrated in a number of studies on a number of pathogens, with functional flagella systems contributing to virulence (Eaton *et al*., 1996; Merino *et al*., 1997; Pratt and Kolter, 1998).

Post-translational modification of proteins by glycosylation was not thought to occur in bacteria until its discovery less than 20 years ago. However, it has been demonstrated that several bacterial proteins, especially surface proteins and flagellins are modified through attachment of N-linked or O-linked carbohydrate (glycan) groups (Logan, 2006; Nothaft and Szymanski, 2010; Iwashkiw *et al*., 2013). O-linked glycosylation of the flagellins has been described for an increasing number of bacteria in both Gram-negative and Gram-positive systems. Gram-negative examples include pathogens such as *Aeromonas* (Tabei *et al*., 2009), *Pseudomonas* (Verma *et al*., 2006), *Campylobacter* (Thibault *et al*., 2001), and *Helicobacter* (Josenhans *et al*., 2002), with Gram-positive pathogen examples including *Clostridium* (Twine *et al*., 2008) and *Listeria* (Schirm *et al*., 2004). Furthermore, the flagellar glycosylation process appears to be essential for the formation and function of an intact flagellar filament, as mutation of genes involved in the biosynthesis of the core glycan results in aflagellated cells (Josenhans *et al*., 2002; McNally *et al*., 2006; Canals *et al*., 2007; Tabei *et al*., 2009). The flagellins of a number of bacterial species including the important pathogens *Helicobacter pylori* and *Campylobacter jejuni* decorate their flagellins in an O-linked manner with the sialic acid-related nonulosonic acid sugars pseudaminic and legionaminic acid (and derivatives) (Goon *et al*., 2003; Schirm *et al*., 2003). Furthermore, the flagella of these species are important colonization factors (Lertsethtakarn *et al*., 2011). The genes required for this flagellin glycosylation process are usually found in extensive genetic loci associated with the flagellin genes termed glycosylation islands. In *C. jejuni* these loci can range from 20 to 50 genes, as these organisms can synthesize both pseudaminic and legionaminic acids and their derivatives in a phase-variable manner (Karlyshev *et al*., 2002). Furthermore, variations in the flagellin glycosylation islands were found to be related to colonization and the strain source from different chicken isolates, with variations in the glycoforms of isolated flagellin (Howard *et al*., 2009). Although the biosynthetic pathways for both pseudaminic and legionaminic acid have been elucidated (Schoenhofen *et al*., 2006; 2009) the pathway of when, where and how flagellin glycosylation takes place is still largely undeciphered, as is the exact identity of the transferase protein that transfers the sugar onto the flagellin. Within the flagellin glycosylation islands of bacteria that decorate their flagellins with nonulosonic acids are a number of genes termed *maf* (motility associated factor). The number of these genes can vary depending upon species and complexity of sugar decoration, ranging for one in *Aeromonas caviae* Sch3 (Parker *et al*., 2012) to seven in some *C. jejuni* strains (Karlyshev *et al*., 2002). The genes encoding Maf proteins are present in the flagellar glycosylation loci of bacteria that glycosylate their flagellins with nonulosonic acids. Maf proteins are believed to be nonulosonic acid-specific glycosyltransferase enzymes not involved in glycan biosynthesis *per se* but acting to transfer activated sugar to flagellin (Guerry *et al*., 2006; Parker *et al*., 2012). In support of this their mutation results in a similar aflagellate phenotype to a glycan biosynthesis mutant (Guerry *et al*., 2006; McNally *et al*., 2006; Parker *et al*., 2012).

In this study the opportunistic human and animal pathogen *Aeromonas caviae* was employed as a model organism to elucidate the flagella glycosylation pathway. *A. caviae* are motile in a liquid environment and motility requires expression of a single polar flagellum that is important for enterocyte adherence (Kirov *et al*., 2004). The polar flagellum filament is made up of two very similar repeating flagellin subunits (FlaA and FlaB) (Rabaan *et al*., 2001). These flagellin monomers are homogenously O-glycosylated with six to eight pseudaminic acid (Pse5Ac7Ac) moieties on serine and threonine residues in the central immunogenic D2/3 domain (Tabei *et al*., 2009), similar to the flagellin of *H. pylori* (Schirm *et al*., 2003). Flagellin glycosylation in *A. caviae* may be considered a prototype or minimal model genetic system since it is encoded by only six genes required for glycosylation of flagellin, while other pathogens such as *C. jejuni* encode many more (between 20 and 50). This is likely due to the fact that *C. jejuni* flagellin is glycosylated with Pse5Ac7Ac and its acetamidino derivative (Pse5Am7Ac), as well as additional glycans including legionaminic acid (Thibault *et al*., 2001), whereas *A. caviae* only utilizes one sugar type. Our aim here was to dissect the flagellin glycosylation, secretion and assembly pathway with a view to further elucidating the order and importance of components such as flagellar chaperones and Maf proteins.

## Results

### Maf1 is required for glycosylation but not secretion of flagellin: unglycosylated flagellin is exported to the culture supernatant

**Fig. 1:**
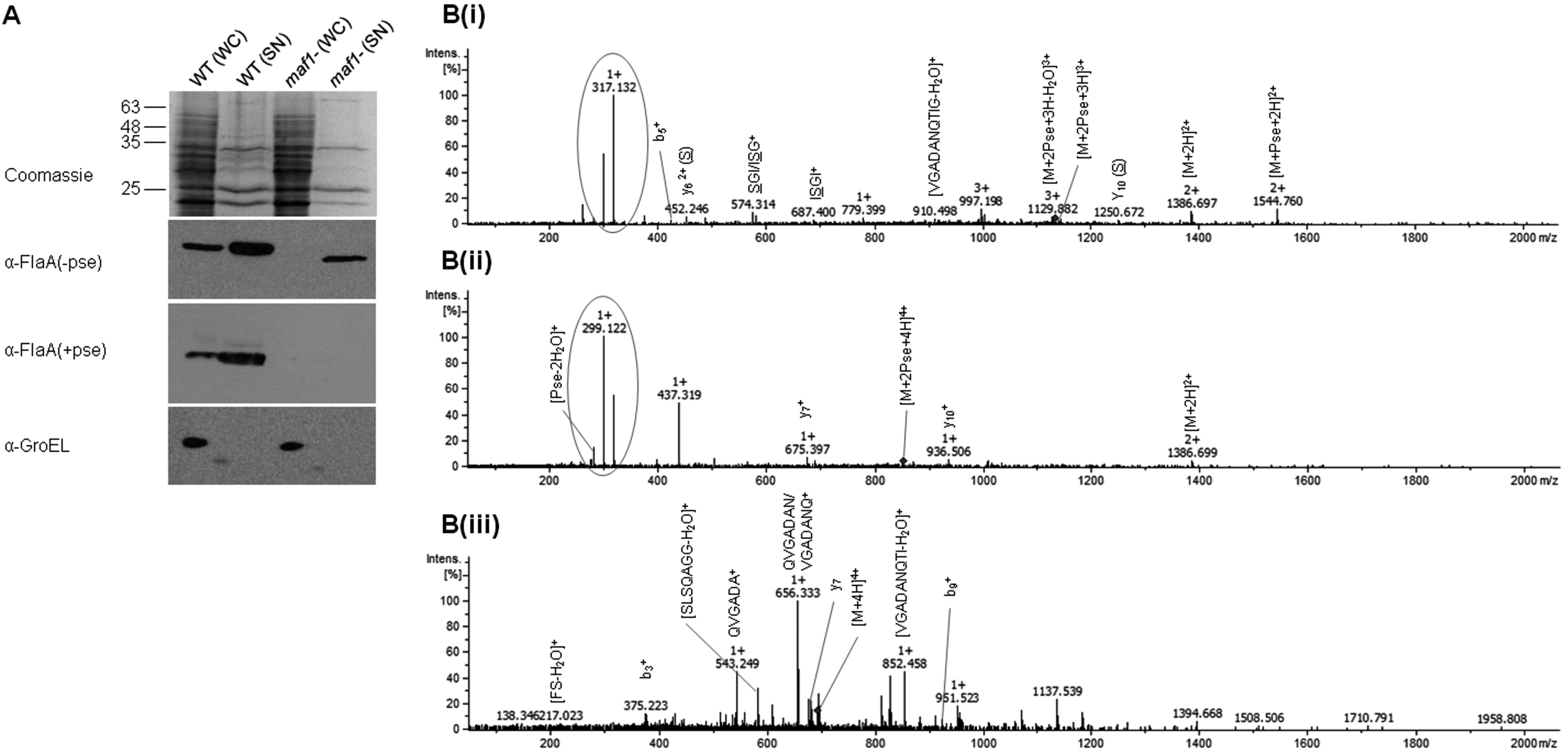
Maf1 is required for flagellin glycosylation.A. Western blot analysis of glycosylated (+pse) and unglycosylated (−pse) FlaA/B using α-FlaA/B(+pse) and α-FlaA/B(−pse) antibodies of whole-cell (WC) preparations and secreted fractions (SN) of *A. caviae* strains wild-type and *maf1* mutant strains. A Coomassie-stained SDS-PAGE gel showing whole-cell preparations and secreted fractions is shown as a loading control. The samples were also probed with α-GroEL as a cell lysis control for secreted fractions and an additional loading control for whole-cell fractions.B. CID tandem mass spectra of the FlaB peptide ^146^FQVGADANQTIGFSLSQAGGFSISGIAK^173^. B(i) MS/MS of the triply charged ion *m/z* 1135.22 that elutes at 96.22 min from wild-type *A. caviae*. The ion corresponds to the peptide containing two Pse residues. B(ii) MS/MS of the quadruply charged ion *m/z* 851.66 that elutes at 96.25 min from wild-type *A. caviae*. The ion corresponds to the peptide containing two Pse residues. B(iii) MS/MS spectra from *A. caviae maf1* mutant flagellin. The quadruply charged ion *m/z* 692.85 which elutes at 74.43 min corresponds to the unglycosylated peptide. S is the potentially modified serine residue.

Using a glycosylated flagellin-specific antibody we have shown that *A. caviae maf1* is required for flagellin glycosylation with no glycosylated flagellin detected in a *maf1* mutant (Parker *et al*., 2012). To investigate this further an antibody was raised against unglycosylated *A. caviae* FlaA flagellin purified from *Escherichia coli* (a system that lacks both pseudaminic acid and the flagellin glycosylation machinery). The antibody generated can recognize both glycosylated and unglycosylated flagellin, as illustrated by the detection of bands of different mobility in Western blots with the smaller band representing the unglycosylated form lacking pseudaminic acid residues in its central section (Fig. 1A). Using these antibodies we demonstrated that glycosylated flagellin is present in both the culture supernatant and whole-cell preparations of the *A. caviae* wild-type strains. In contrast, the unglycosylated flagellin produced by the *maf1* mutant could only be detected in the culture supernatant, at lower levels than that of the wild-type glycosylated flagellins. In contrast, the intracellular levels of unglycosylated flagellin were too low to detect using our methodology (Fig. 1A). To control for cell lysis or any escape of cytoplasmic proteins in to the secreted fraction, immunoblots were performed using an antibody against the cytoplasmic chaperone protein GroEL on the same samples as for the unglycosylated flagellin antibody. GroEL is a ubiquitous cytoplasmic chaperonin in bacteria (Jyot *et al*., 1999) that is a well-established cytoplasmic marker (Tapia-Pastrana *et al*., 2012). These anti-GroEL immunoblots were negative for the supernatants while a control lane using whole *A. caviae* cells was positive indicating no cell lysis had occurred in these samples.

To further support the notion that we had detected unglycosylated flagellin in the secreted fractions, we analysed the flagellin isolated from the precipitated culture supernatant of a *maf1* mutant strain and the glycosylated flagellin sheared from the surface of wild-type *A. caviae,* by mass spectrometry. For isolation of glycosylated flagellin, *A. caviae* was grown on TSB agar to reduce shearing forces exhibited in shaking liquid culture, while obtaining more material than was possible from equivalent standing liquid cultures. Flagellin samples underwent in-gel trypsin digestion followed by reverse-phase liquid chromatography (LC) coupled to MS analysis. Tandem mass spectrometric (MS/MS) using collision-induced dissociation (CID) was acquired and data were interrogated using EasyProt (Gluck *et al*., 2013). Input of MS data into EasyProt showed wild-type *A. caviae* flagellins were modified with pseudaminic acid while only unglycosylated flagellin peptides could be identified for the *maf1* mutant. For confirmation, manual inspection of raw data was also performed using both inspection of diagnostic ions typical of pseudaminic acid in the low m/z region of the tandem mass spectra, and the mass of precursor/fragment ions predicted from *in silico* digests using the protein product tool on the Protein Prospector website (http://prospector.ucsf.edu/prospector/mshome.htm). Peptides modified with pseudaminic acid that undergo CID MS/MS, release the sugar moiety as a preferential fragmentation channel during the fragmentation process, generating ions at *m/z* 317.13 and 299.12 which correspond to the pseudaminic acid oxonium ion and its dehydrated form respectively (Thibault *et al*., 2001). These signature ions are absent in the MS/MS spectra, indicating the absence of the modification [Fig. 1B(iii)]. In addition, due to the size and charge states of the identified glycopeptides [Fig. 1B(i)(ii)] and the peptides in their unmodified form [Fig. 1B(iii)], typical y and b ions were not found to be predominant in the MS/MS spectra. The major fragments observed are intact peptide ions and some internal ions (Fig. 1B). These MS data indicate that wild-type *A. caviae* flagellin presents pseudaminic acid as a post-translational modification while *maf1* mutant flagellin is unglycosylated.

To our knowledge, this is the first example of the export of unglycosylated flagellin to the culture medium, and is in contrast to previous studies that have suggested unmodified flagellin is trapped in the cytoplasm (Josenhans *et al*., 2002). These data suggest that unglycosylated flagellin is still export competent, i.e. recognized by the export apparatus of the flagellar T3SS (FT3SS) and thus contains a functional secretion signal and ability to dock and engage the export apparatus, but also that the presence of pseudaminic acid glycosylation in the D2/D3 domain is not required for passage through the FT3SS.

### Motility-associated factor (Maf) directly interacts with flagellin *in vivo*

As mutation of the *A. caviae maf1* gene causes loss of glycosylation from the flagellin protein resulting in a lack of motility, and since we hypothesize that Maf1 is a putative pseudaminyltransferase we wished to investigate whether it might act via a direct interaction with flagellin subunits.

**Fig. 2:**
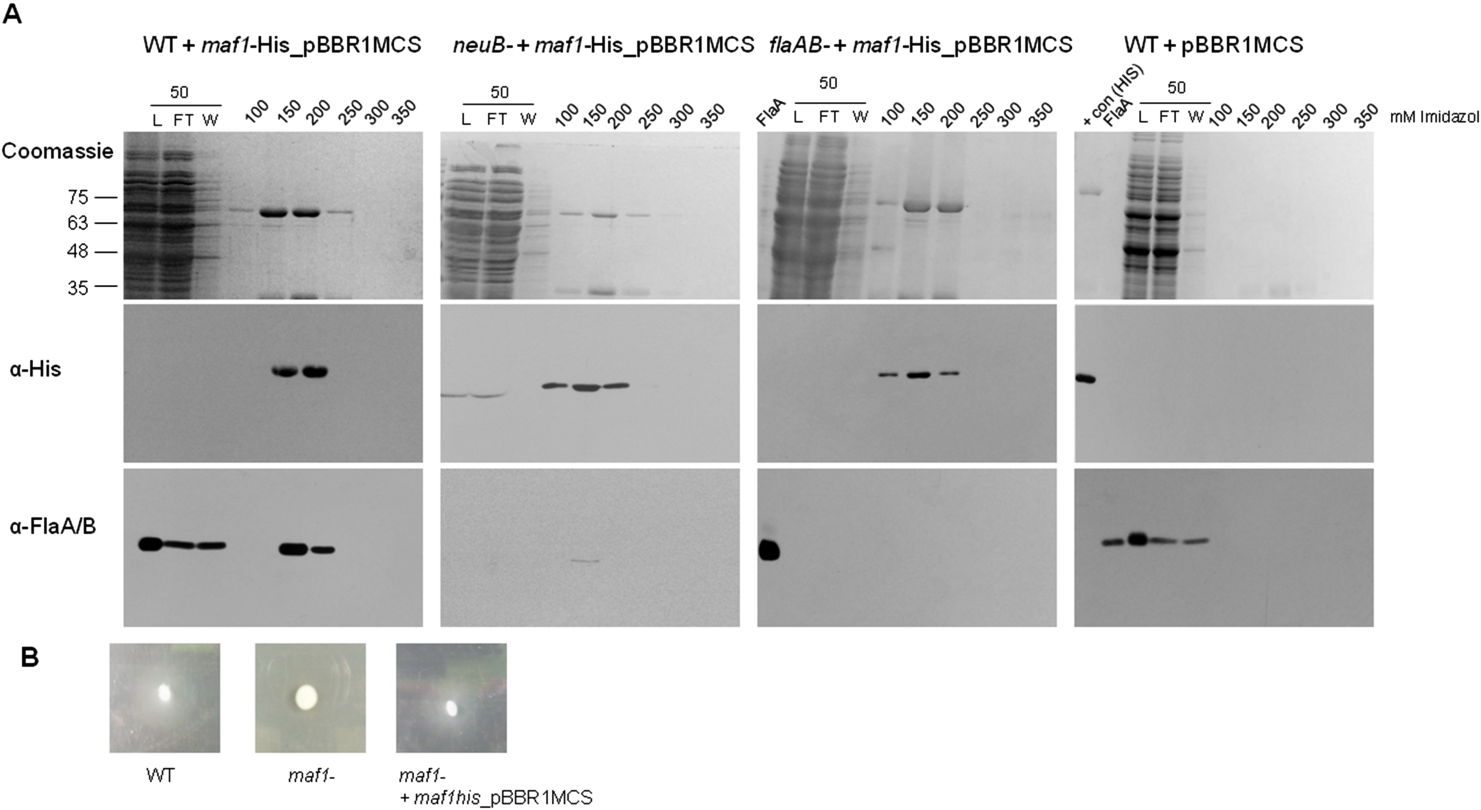
Flagellin directly interacts with Maf1 *in vivo.*A. Coomassie-stained SDS-PAGE gels (top panel) of co-purified Maf1-His and FlaA/B from *A. caviae* wild-type (far left); *neuB*-harbouring pBBR1MCS*-maf1his* (centre left); *flaAB* mutant harbouring pBBR1MCS*-maf1his* (centre right); and wild-type harbouring empty pBBR1MCS (far right); purified by nickel affinity chromatography. Western blot analysis of resulting purification fractions using α-His antibodies (middle panel) and α-FlaA/B(−pse) antibodies (lower panel). The load (L), flowthrough (FT) and wash (W) fractions were also analysed for the presence of Maf1-His and FlaA/B.B. Motility of *A. caviae* wild-type, *maf1* mutant and *maf1* mutant containing pBBR1MCS expressing *maf1-his* inoculated on 0.25% semisolid bacteriological agar.

After several attempts to purify the *A. caviae* Maf1 using common *E. coli* protein over-production strains and plasmids, levels of IPTG, growth temperatures etc. we discovered that full-length *A. caviae* Maf1 appeared to be toxic to *E. coli*. Therefore an alternative approach was then taken to obtain high levels of purified Maf1. In our previous work we complemented the non-motile phenotype of *A. caviae maf1* mutant by introducing the wild-type copy of *maf1* with approximately 170 bp of upstream sequence (predicted to encompass its native promoter) (Parker *et al*., 2012) and which we assume due to the lack of toxicity does not function in *E. coli,* on the multicopy broad-host-range mobilizable vector pBBR1MCS (Cm^r^). This *maf1* plasmid was mutated to introduce the codons encoding a C-terminal penta his-tag into the *maf1* construct, allowing its purification via nickel affinity chromatography when expressed. Using motility assays carried out on 0.25% bacteriological agar, we showed that the addition of the penta-his tag did not affect the function of *maf1* since this construct was able to fully complement the non-motile phenotype of the *A. caviae maf1* mutant strain (Fig. 2B).

To begin to investigate whether Maf1-His might interact with flagellin monomers in *in vivo* pull-down experiments the *maf1-his* plasmid construct was introduced into wild-type *A. caviae*. Maf1-His (and any associated proteins) were then purified by nickel-affinity chromatography and all resulting fractions were analysed for the presence of flagellin by Western blotting using our anti-flagellin antibody that is able to recognize both glycosylated and unglycosylated flagellin. Where Maf1-His was purified from wild-type *A. caviae* harbouring the pBBR1MCS*-maf1his* construct, the flagellins FlaAB co-purified with Maf1-His, and were observed in fractions containing high levels of Maf1-His (Fig. 2A, far left panel). In order to ascertain whether Maf1 would also interact with unglycosylated flagellin, i.e. its original substrate, the construct was introduced into an *A. caviae neuB* mutant strain that is unable to synthesize pseudaminic acid and so would lack activated pseudaminic acid that is used as the source of sugar that is transferred onto the flagellin proteins (Tabei *et al*., 2009). In this pseudaminic acid devoid *A. caviae* strain, the flagellins FlaAB still co-purified with Maf1, however at lower amounts (Fig. 2A centre left panel). This observation was probably due to the largely insoluble nature of unglycosylated flagellin as FlaAB levels are low in a *neuB* mutant background (data not shown); however, this result indicates that the presence of pseudaminic acid is not required for the binding of Maf1 to the flagellins FlaAB. Negative control experiments using an *A. caviae* mutant strain lacking the two flagellin monomers *flaA* and *flaB* (*flaAB*) and wild-type *A. caviae* harbouring only the empty pBBR1MCS showed that the flagellins were not observed in the corresponding purifications fractions (Fig. 2A centre right and far right panels respectively) and were dependent on the presence of both Maf1 and FlaA/B. This is the first example of the successful purification of any Maf protein, as well as the first unequivocal evidence for a direct *in vivo* interaction between flagellin and Maf, which we propose is a putative flagellin glycosyltransferase.

### Glycosylation of flagellin occurs in the cytoplasm, independently of the flagellin-specific chaperone FlaJ and polymerization of the filament

The formation of a functional flagella filament and therefore swimming motility has previously been shown to be dependent on the presence of the flagellin-specific chaperone *flaJ* (*fliS* in *E. coli* and *Salmonella*) (Bennett and Hughes, 2000), and we have shown that mutation of *flaJ* in *A. caviae* results in non-motile cells, that lack flagella (Rabaan *et al*., 2001). We now wished to examine the possible role of the flagellin-specific chaperone FlaJ in this process and pathway, i.e. to try and examine if it is required for glycosylation to occur or whether its role is strictly related to its role as an export pilot and chaperone.

**Fig. 3:**
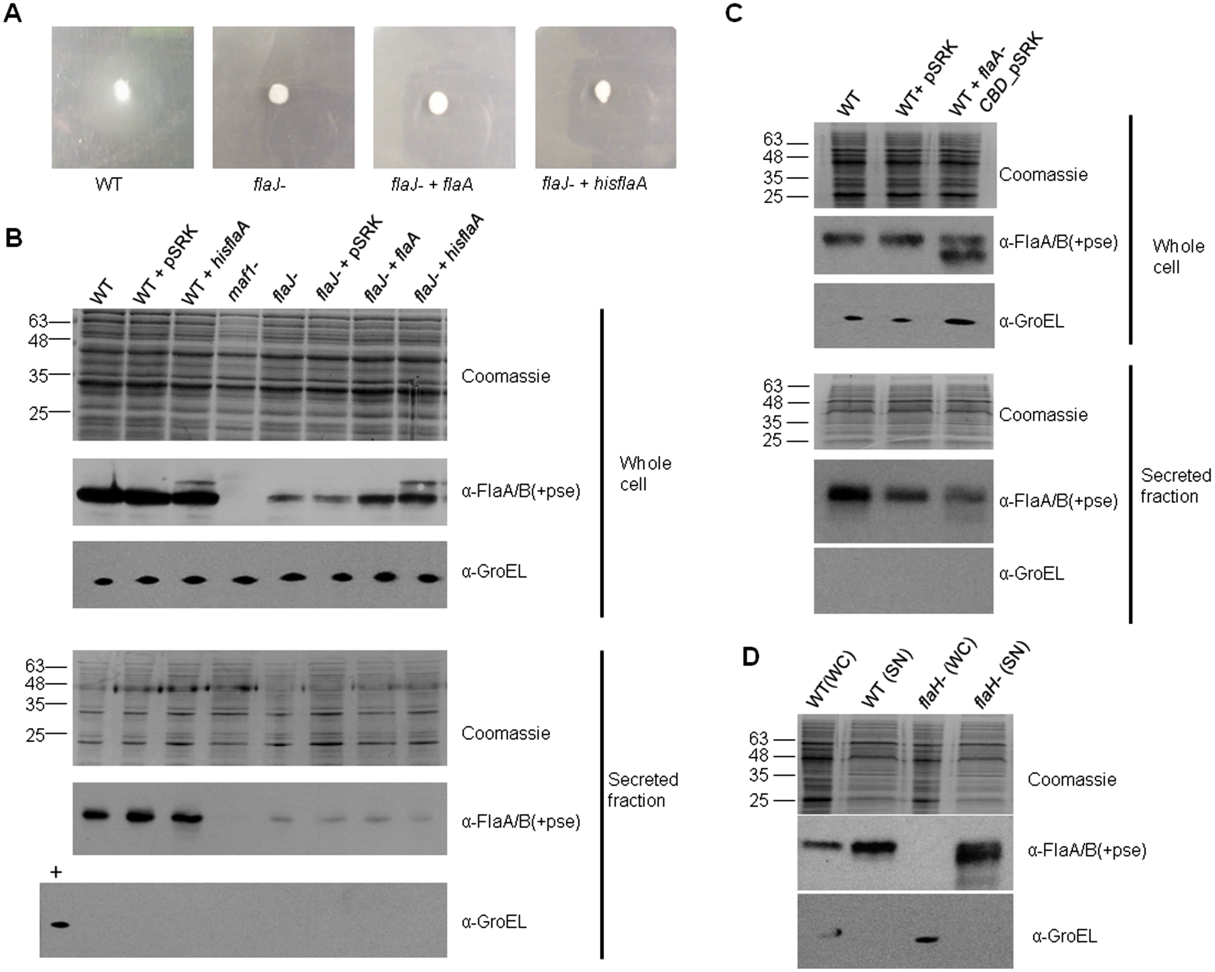
FlaJ is not required for flagellin glycosylation but is required for export.A. Motility of *A. caviae* wild-type, *flaJ* mutant, *flaJ* mutant harbouring pSRK *flaA*, and *flaJ* mutant harbouring pSRK_*hisflaA* inoculated on 0.25% semisolid bacteriological agar.B. Western blots of FlaA/B(+pse) using α-FlaA/B(+pse) antibodies of whole-cell preparations and secreted fractions of *A. caviae* strains (wild-type, wild-type + pSRK, wild-type + pSRK_*hisflaA*, *maf1* mutant, *flaJ* mutant, *flaJ* mutant + pSRK, *flaJ* mutant + pSRK_*flaA*, and *flaJ* mutant + pSRK_*hisflaA*). Coomassie-stained SDS-PAGE gels showing whole-cell preparations and secreted fractions are given as a loading control. The samples were also probed with α-GroEL as a cell lysis control for secreted fractions and an additional loading control for whole-cell fractions.C. Western blots of FlaA/B(+pse) using α-FlaA/B(+pse) antibodies of whole-cell preparations and secreted fractions of *A. caviae* strains (wild-type, wild-type + pSRK, wild-type + pSRK_*flaA-CBD*), Coomassie-stained SDS-PAGE gels showing whole-cell preparations and secreted fractions are given as a loading control. The samples were also probed with α-GroEL as a cell lysis control for secreted fractions and an additional loading control for whole-cell fractions.D. Western blots of FlaA/B(+pse) using α-FlaA/B(+pse) antibodies of whole-cell (WC) preparations and secreted fractions (SN) of *A. caviae* wild-type and *flaH* mutant strains. A Coomassie-stained SDS-PAGE gel showing whole-cell preparations and secreted fractions is given as a loading control. The samples were also probed with α-GroEL as a cell lysis control for secreted fractions and an additional loading control for whole-cell fractions.

As a first step to examining this question we designed an experiment to confirm the absolute reliance on FlaJ for export, by overexpressing *A. caviae* FlaA flagellin in a *flaJ* mutant background to test if motility and secretion could be restored to any detectable level. Motility assays revealed that overexpression of either FlaA or His-FlaA (which is also able to be glycosylated) in an *A. caviae flaJ* mutant was unable to restore motility (Fig. 3A).

To investigate whether interaction with the flagellin-specific export chaperone FlaJ is required for flagellin glycosylation as well as protein export, we investigated the glycosylation and export status of flagellin in an *A. caviae flaJ* mutant strain by Western blotting using an antibody that recognizes *A. caviae* glycosylated flagellin only (Fig. 3B). Small amounts of glycosylated flagellin could be observed in whole-cell preparations of an *A. caviae flaJ* mutant, indicating FlaJ is not essential for the glycosylation process. This was further confirmed when *A. caviae* flagellin FlaA and His-FlaA were overexpressed in a *flaJ* mutant strain, as a result levels of glycosylated flagellin in the whole-cell preparations increased markedly. It was also noted that His-FlaA while successfully glycosylated was located intracellularly and not observed in secreted fractions (Fig. 3B), most likely due to the presence of the N-terminal his-tag blocking the functionality of the N-terminal signal sequence. Intriguingly, overexpression of His-FlaA in an *A. caviae flaJ* mutant also increased levels of glycosylated untagged flagellin in whole-cell preparations. It is possible that these increased levels of glycosylated untagged flagellin may be degraded His-FlaA that lack the His-tag. Despite these increased levels of intracellular glycosylated flagellin, swimming motility was not restored (Fig. 3A).

**Fig. 4:**
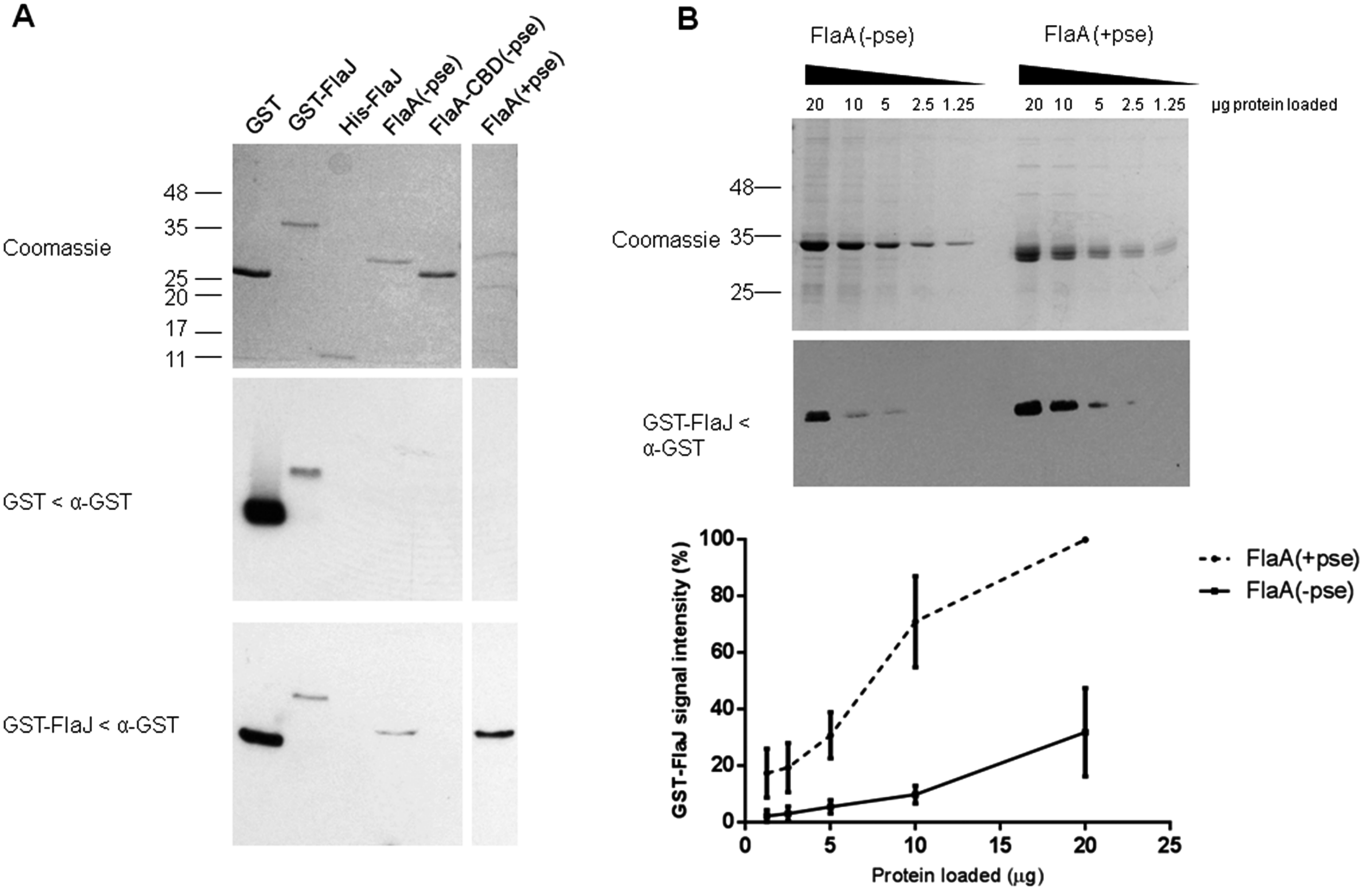
FlaJ shows a stronger affinity for FlaA (+pse) than FlaA (−pse).A. Coomassie-stained SDS-PAGE gel and far Western blots of GST, GST-FlaJ, His-FlaJ, FlaA (−pse), FlaA-CBD (−pse), and FlaA (+pse) probed with purified GST (middle panel) or GST-FlaJ (lower panel) and α-GST antibodies.B. Coomassie-stained SDS-PAGE gel and representative far Western blot of FlaA (−pse), and FlaA (+pse) probed with purified GST-FlaJ and α-GST antibodies. Blots were performed in triplicate and the average signal density corresponding to deposited GST-FlaJ interacting with FlaA (−pse), and FlaA (+pse) was plotted as a % with the highest density band as 100%.

When the secreted protein fractions were harvested from the culture supernatants of various strains by precipitation and levels of exported flagellins assayed we observed that very low levels of flagellin were observed in the supernatants of all the *A. caviae flaJ* mutant strains including those that were overexpressing FlaA, indicating that *flaJ* is optimal for the export of the flagellin (Fig. 3B). Following this observation that the chaperone was not required for glycosylation, a truncated version of the *A. caviae flaA* gene lacking the sequence encoding the C-terminal chaperone-binding domain (CBD amino acids 261–306) was expressed from the broad-host inducible vector pSRKGm in the *A. caviae* wild-type strain. The C-terminal chaperone-binding domain of flagellin is a helical region required for the binding of flagellin to its cognate chaperone, promoting its stability and preventing intracellular *in vivo* polymerization of flagellin (Auvray *et al*., 2001). Western blot analysis showed that the truncated version of the flagellin (FlaA-CBD, which we show below is also unable to bind FlaJ in *in vitro* assays, Fig. 4A) was observed in whole-cell preparations in its glycosylated form, although no FlaA-CBD could be observed in the secreted fractions. Taken together these data indicate that like the flagellin-specific chaperone FlaJ, the chaperone-binding domain of FlaA is not required for glycosylation of flagellin, i.e. Maf must still be able to interact with the FlaA-CBD construct. In parallel we also performed Anti–GroEL Western blots, as cell lysis controls (Fig. 3B and C). These data, confirm unequivocally that glycosylation of flagellin occurs in the cytoplasm prior to export via its dedicated type three secretion system (T3SS).

In addition to the role of FlaJ, the role of FlaH in flagellin glycosylation was investigated. The *flaH* gene encodes a 464-amino-acid protein (48.8 kDa) that acts as a flagellar cap protein (FliD in *E. coli* and *Salmonella*). The FlaH cap is required for the formation of a fully functional filament and is present at the distal growing tip of the filament and is required for efficient polymerization of flagellins. In the absence of FlaH, flagellin monomers are secreted in to the culture supernatant of liquid grown cultures (Yonekura *et al*., 2000). Here we investigated the glycosylation status of these secreted flagellin monomers isolated from an *A. caviae flaH* mutant strain. Using the antibody that recognizes *A. caviae* glycosylated flagellin only, large amounts of glycosylated flagellin could be observed in the secreted fraction of the *A. caviae flaH* mutant strain, with very little visible in the whole-cell preparation (Fig. 3D). As with all of the other secreted fraction immunoblots in this study, anti-GroEL Western blots were carried out as a control to ensure that the glycosylated flagellin identified in secreted fraction of the *A. caviae flaH* mutant strain was not due to cell lysis. Here we show that an *A. caviae* flagella cap *flaH* (*fliD*) mutant that lacks the presence of a fully functional filament, but is still able to export flagellin, glycosylates flagellin to wild-type levels and that no feedback mechanisms are in place to prevent the glycosylation of flagellin in the absence of flagellar filament formation.

### The flagellin-specific chaperone FlaJ interacts with unglycosylated and glycosylated flagellin

We have shown that glycosylation occurs in the cytoplasm independently of FlaJ (flagellin-specific chaperone), and unglycosylated flagellin appears to be exported at lower levels than the glycosylated version, where FlaJ is still present (in the *maf* mutant strain). In response to these findings, we wished to assess the binding specificities of the *A. caviae* flagellin-specific chaperone FlaJ with *A. caviae* flagellin using far Western blots with GST-FlaJ as a probe (Fig. 4). Far Western blots showed that GST-FlaJ directly interacted with unglycosylated FlaA (purified from *E. coli*) and glycosylated FlaA purified from an *A. caviae flaB* mutant *in vitro*, but, as expected, GST-FlaJ failed to interact with a truncated form of unglycosylated FlaA flagellin that lacked the chaperone-binding domain (amino acids 261–306), i.e. the FlaJ binding site (Fig. 4A). The FlaJ−flagellin interaction observed was dependent upon the presence of FlaJ, with no interaction observed when GST alone was used as a probe. The far Western also showed that GST-FlaJ did not interact with His-FlaJ, supporting previous work that showed the flagellin-specific chaperone functions as a monomer (Evdokimov *et al*., 2003), at least in the absence of an interacting partner. Over a number of repeats, a stronger signal was always observed for the binding of GST-FlaJ to the glycosylated version of the *A. caviae* FlaA flagellin than to the unglycosylated version. Therefore, this was further investigated in an experiment were both glycosylated and unglycosylated flagellins were titrated across an SDS-PAGE gel and transferred to nitrocellulose before being probed again with a constant concentration of GST-FlaJ in far Western blots (Fig. 4B). Results showed that GST-FlaJ binds the glycosylated version of FlaA with a stronger affinity than unglycosylated version as illustrated by the interaction of larger amounts of GST-FlaJ (approximately 5× more) with equimolar amounts of glycosylated flagellin than unglycosylated flagellin (Fig. 4B).

**Fig. 5:**
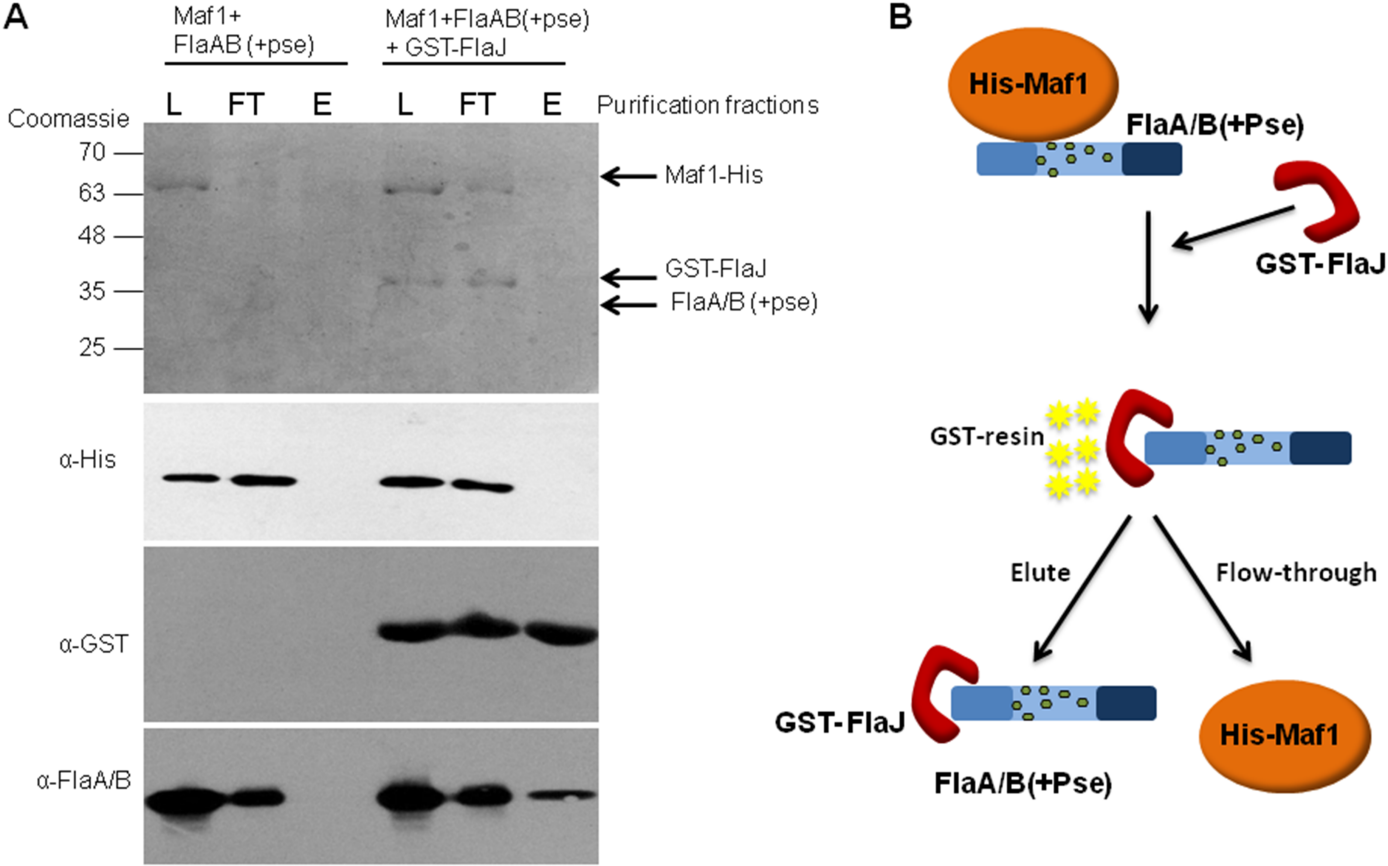
Flagellin glycosylation occurs prior to chaperone binding.A. Coomassie-stained SDS-PAGE gel and Western blots of FlaA/B ‘handover’ event. Western blots of Maf1-His, GST-FlaJ and FlaA/B(+pse) using α-His, α-GST and α-FlaA/B(+pse) antibodies respectively, were analysed in the load (L) flowthrough (FT) and elution (E) purification fractions.B. Schematic showing overview of ‘handover’ event. The co-purified Maf1-His and FlaA/B complex was mixed with GST-FlaJ. The protein mixture was subjected to glutathione affinity chromatography. FlaA/B dissociates from His-Maf1 and binds GST-FlaJ, which subsequently binds the GST-resin. Maf1 is removed from the protein mixture in the flowthrough and during the wash stages. FlaA/B and GST-FlaJ are co-purified and released from the resin in the elution fraction.

A possible biological reason for this would be that flagellin glycosylation occurs prior to chaperone binding for the efficient export of flagellin. To further investigate this section of the flagellin glycosylation pathway, the *A. caviae* Maf1–FlaAB complex purified from wild-type *A. caviae* in the pull-down assay shown in Fig. 2 was mixed with equimolar amounts of purified GST-FlaJ *in vitro* and the protein mixture was subjected to glutathione affinity chromatography. Western blots of the resulting eluted fractions showed that flagellin co-eluted from the glutathione sepharose resin with GST-FlaJ, whereas all of the Maf1 was found in the flow-through. In contrast, in the absence of FlaJ all of the flagellin was found in the flow-through, with none observed in the eluted fraction (Fig. 5). This suggests that FlaJ captures glycosylated flagellin from Maf1 in a hand-over reaction that might mimic events *in vivo* where Maf1 glycosylates FlaA/B before passing it over to the FT3SS pathway chaperone FlaJ for export. Taken together these data strongly indicate that glycosylation of flagellin takes place in a Maf1-dependent manner in the cytoplasm prior to chaperone binding.

## Discussion

*Aeromonas caviae* strain Sch3 appears to have the simplest O-linked flagellar glycosylation island that has been identified to date (Tabei *et al*., 2009; Parker *et al*., 2012). This island is composed of five genes encoding the enzymes required for the biosynthesis of CMP-Pse5Ac7Ac and a single *maf* gene potentially encoding the glycosyltransferase, it is therefore an ideal model to investigate the fundamental mechanism and pathway of bacterial flagellin glycosylation. In this study, we have shown that unglycosylated flagellin has a reduced mass compared its glycosylated counterpart and have demonstrated that this corresponded to the loss of Pse5Ac7Ac from the flagellin in an *A. caviae maf1* mutant by Mass spectrometry. However, unlike other studies that indicate unglycosylated flagellins are present in whole-cell preparations we have shown for the first time the presence of unglycosylated flagellin in the secreted fraction and that it is not trapped in the cytoplasm as previously thought (Josenhans *et al*., 2002; Goon *et al*., 2003). We postulate that these differences may be due to previous studies being unable to detect the reduced amounts of flagellin secreted in *maf* strains as demonstrated in this study. Our evidence proves for the first time that unglycosylated flagellin is capable of being exported by the FT3SS suggesting that the presence of glycans in the central D2/D3 domains does not function as a secretion signal in this secretion system. Although unglycosylated flagellin was exported, this appeared to be at a much lower level than the *A. caviae* wild-type strain. Therefore we extensively investigated the role of the *A. caviae* flagellin-specific chaperone FlaJ in the flagellin glycosylation process. In a number of bacteria this has been shown to play a major role in the export of flagellin, and where the absence of FlaJ (FliS) results in aflagellated non-motile cells (Rabaan *et al*., 2001). The current model for flagellin export is that flagellin is bound by the specific chaperone FlaJ, at its C-terminal chaperone-binding domain and that the chaperone maintains the flagellin in an unfolded form, stabilizing it and preventing its polymerization in the cytoplasm (Auvray *et al*., 2001). At this stage the flagellin is piloted by FlaJ (FliS) to the flagellar export apparatus housed within the basal body, where it is docked for the initial stage of export (Akeda and Galan, 2005; Evans *et al*., 2006; Paul *et al*., 2008).

Previous studies have suggested that the process of flagellin glycosylation occurs prior to (Ewing *et al*., 2009) or possibly coupled to export (Logan, 2006). Here, using the *A. caviae flaJ* mutant and the flagellin lacking the chaperone-binding domain (CBD) we have unequivocally proved that FlaJ and the CBD are essential for flagellin export, but flagellin glycosylation occurs independently of the chaperone FlaJ and the CBD of flagellin. Glycosylated flagellin can be observed in a *flaJ* mutant or in the absence of the flagellin's CBD. These data therefore demonstrate that glycosylation occurs in the cytoplasm prior to export. This is in agreement with data from *C. jejuni* 81-176 showing that flagellin glycosylation can occur in the absence of a functional flagellar export system where the presence of glycosylated flagellin was observed in whole-cell preparations of strains mutated in genes encoding basal body and hook proteins required for flagellin export and assembly (Ewing *et al*., 2009).

**Fig. 6:**
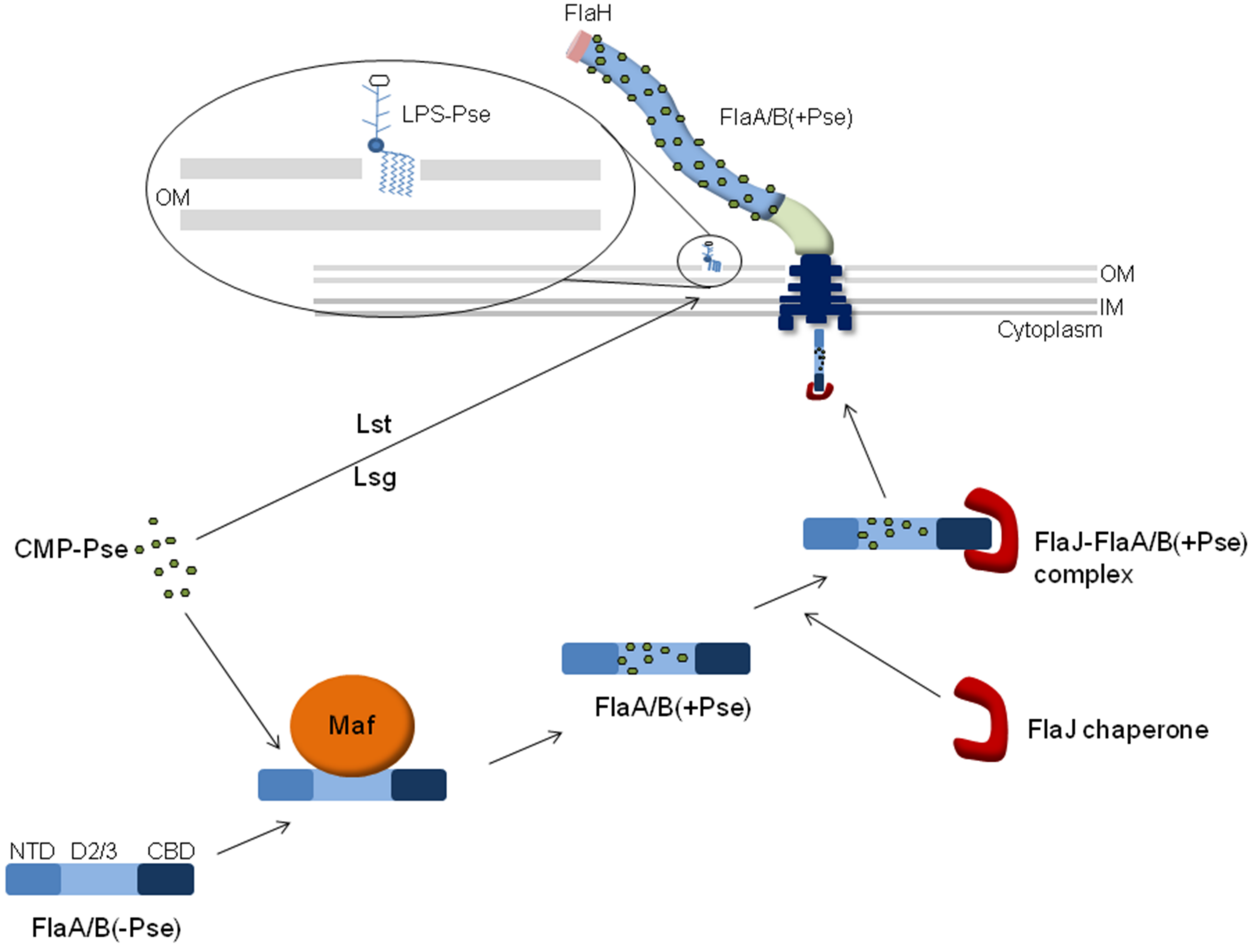
Model depicting the flagellin glycosylation and export pathway. Activated pseudaminic acid (CMP-Pse) either is transferred onto a sugar-antigen carrier lipid (ACL) by Lst to create an LPS O-antigen unit, which is subsequently transported across the cytoplasmic membrane by Lsg, or is transferred on to the central D2/3 domain of unglycosylated flagellin the cytoplasm in a Maf dependant manner. Glycosylated flagellin is then bound by the flagellin-specific chaperone FlaJ, which is dependent on presence of C-terminal chaperone-binding domain (CBD) and piloted to the basal body of the flagellar where it is exported via it's dedicated T3SS in an unfolded form. For polymerization, the flagellin is folded, exposing the central D2/3 domain and attached glycans along the surface of the filament. The cytoplasmic glycosylation process occurs independently of the presence of the flagellar cap FlaH and a functional flagellar filament.

We have also shown that FlaJ binds glycosylated flagellin with a higher affinity than unglycosylated flagellin, and that this binding allows export to occur more efficiently. This explains why lower amounts of unglycosylated flagellin were found in secreted fractions of the *maf1* mutant compared to the glycosylated form secreted from *A. caviae* wild-type. One explanation for this would be that in the absence of FlaJ, flagellin is translated and glycosylated in the cytoplasm but without FlaJ to bind glycosylated flagellin and maintain its unfolded form, most of the flagellin degrades and none is successfully exported to form a filament. Conversely, in the absence of Maf, flagellin remains unglycosylated with smaller amounts binding FlaJ, but these smaller amounts are able to be successfully exported. The exported unglycosylated flagellin (in a *maf* mutant strain) is unable to form a functional filament and is found in reduced amounts (compared to wild-type glycosylated flagellin) in the culture supernatant (Fig. 6). Our data also suggest that the FT3SS in species that glycosylate their flagellin have evolved to make this process more efficient since chaperone recognition of the glycosylated flagellin is preferential to its non-glycosylated form, promoting efficient export of the glycosylated flagellin over unglycosylated flagellin. In support of this notion it is notable that in neither a wild-type or *flaH* mutant strain (that secretes monomeric flagellin), one never observes secreted unglycosylated flagellin (Figs 1A and 3D).

Using our *A. caviae* model system, we have shown for the first time using protein–protein interactions that Maf and flagellin directly interact *in vivo* in the cytoplasm of *Aeromonas*, reinforcing the hypothesis that Maf proteins are the glycosyltransferase enzymes responsible for transferring activated sugars on to flagellins via O-linked glycosylation (Ewing *et al*., 2009; Parker *et al*., 2012). Furthermore, flagellin can be ‘handed over’ from Maf to the chaperone FlaJ, when a Maf–flagellin complex is mixed with chaperone. This then results in a FlaJ−flagellin complex with the release of free Maf which may then be recycled to glycosylate a fresh flagellin subunit. Based on previous evidence and our observation that GST-FlaJ does not self-interact with FlaJ-His in far Westerns, we surmise this is a heterodimeric complex comprising a 1:1 complex of FlaJ : FlaA (Evdokimov *et al*., 2003; Muskotal *et al*., 2006).

Based on the evidence gathered in this article we propose a modified model of the flagella glycosylation and secretion process (Fig. 6). In summary, we propose that flagellin is glycosylated by the flagellin glycosyltransferase Maf1 via a direct interaction between the two proteins that occurs independently of the activated sugar substrate (CMP-Pse). Following glycosylation, glycosylated flagellin is bound and handed over to the flagellin-specific chaperone FlaJ via its C-terminal chaperone-binding domain for which it has a higher affinity than the unglycosylated version. Maf1 is then released for further rounds of flagellin glycosylation. The FlaJ chaperone-flagellin complex is then delivered to the dedicated T3SS through piloting by FlaJ (FliS) to the flagellar export apparatus within the basal body. After docking, flagellins are unfolded by the FT3SS ATPase and fed into the 2 nm (20 Å) FT3SS lumen in an extended unfolded form, (Blocker *et al*., 2001; Yonekura *et al*., 2003). The O-linked sugar moieties of the glycosylated flagellin are unlikely to be too big to allow export through the relatively narrow pore of its dedicated T3SS due to their monomeric nature (Tabei *et al*., 2009). The unfolded glycosylated flagellins are exported and in the presence of the FlaH cap are able to polymerize at the distal tip, forming a functional flagellar filament.

One intriguing aspect of our observations is the finding that FlaJ displaces Maf binding of flagellin in our *in vitro* handover experiment although we would propose that their binding sites differ, with the C-terminal CBD being essential for FlaJ binding but not required for Maf dependant glycosylation. Also, how the flagellin-specific chaperone senses the glycosylation status of its substrate while binding an unglycosylated region remains an interesting question. One thing we have considered but been unable to test due to technical difficulties with highly insoluble unglycosylated flagellin, is whether Maf in a complex with unglycosylated flagellin (from the *neuB* mutant strain) can be displaced by FlaJ. We would predict that given the lower affinity of the chaperone for unglycosylated flagellin, this handover event would not occur so readily.

Overall, this article provides the first real evidence-based insights into the series of events occurring during the flagellin glycosylation pathway in pathogenic bacteria. Our data also highlight that it is likely that in those species that glycosylate flagellin there seems to be a major structural role for the glycan addition that seems to influence polymerization ability rather than act as a secretion signal. What ultimate function the glycan modification really serves is unclear at this point. It may play a role in antigenic recognition in infection and is certainly a source of antigenic variation in some species such as *Pseudomonas aeruginosa* and *C. jejuni*. Studies have shown that *Pseudomonas* flagellin glycosylation aids TLR-5 response (Hayashi *et al*., 2001), inducing the production of the pro-inflammatory cytokine, IL-8, and this IL-8 release is considerably reduced in response to unglycosylated mutant flagellins (Verma *et al*., 2005). These questions are actively being studied in our group and remain exciting questions for the future.

## Experimental procedures

### Bacterial strains, plasmids, and growth conditions

Plasmids and strains used in this study are described in Table 1. Oligonucleotides are described in Table S1. Where required, DNA restriction endonucleases, T4 DNA ligase, and alkaline phosphatase were used as recommended by the suppliers (New England Biolabs, USA). *E. coli* strains were grown in Luria–Bertani (LB) Miller broth and on LB Miller agar, while *A. caviae* strains were grown in brain heart infusion broth (BHIB) or on Columbia blood agar (Oxoid). For harvesting glycosylated flagellin, *A. caviae* strains were grown on TSB agar. Growth of *E. coli* and *A. caviae* strains was typically carried out at 37°C. Ampicillin (50 μg ml^−1^), nalidixic acid (50 μg ml^−1^), kanamycin (50 μg ml^−1^), gentamicin (25 μg ml^−1^), streptomycin (50 μg ml^−1^), and chloramphenicol (25 μg ml^−1^) were added to the different media when necessary for selection.

**Table 1:** Strains and plasmids used in this study

Strain or plasmid	Genotype and use or description	Source or reference
*E. coli* strains		
DH5α	F^−^ Phi80*dlacZ* ΔM15 Δ(*lacZYA-argF*)U169 *deoR recA1 endA1* *hsdR17*(rK−mK+) *phoA supE44* lambda- *thi*-1; used for general cloning/plasmid maintenance	Invitrogen
BL21(DE3)	F^−^ *ompT hsdSB*(r_B_^−^ m_B_^−^) *gal dcm* (DE3); used to express recombinant proteins in *E. coli*	Laboratory stock
S17-1λ*pir*	*hsdR pro recA*, RP4-2 in chromosome, Km::Tn*7* (Tc::Mu) λ*pir*, Tp^r^ Sm^r^; used for conjugative transfer	de Lorenzo *et al*. (1990)
*Aeromonas* strains		
*A. caviae* Sch3N	Sch, spontaneous Nal^r^	Gryllos *et al*. (2001)
*A. caviae* AAR8	Sch3N; *flaJ*::km^r^	Rabaan *et al*. (2001)
*A. caviae* JPS01	Sch3N; *maf1*::km^r^	Parker *et al*. (2012)
*A. caviae* AAR59	Sch3N; *flaH*::km^r^	Rabaan *et al*. (2001)
*A. caviae* SMT166	Sch3N; *neuB*::km^r^	Tabei *et al*. (2009)
*A. caviae* AAR31	Sch3N; *flaA*::Cm^r^, *flaB*::Km^r^	Rabaan *et al*. (2001)
*A. caviae* AAR27	Sch3N; *flaB*::Km^r^	Rabaan *et al*. (2001)
Plasmids		
pGEM	Cloning vector, Amp^r^	Promega
pET28a	Expression vector with hexa-histidine tag, Km^r^	Novagen
pGEX4T3	Expression vector with GST tag, Amp^r^	GE Healthcare
pBBR1MCS	Broad-host-range vector, IncP, -W, -Q, ColE1 and p15A compatible, contains pBluescript IIKS-*lacZα*-polylinker, Cm^r^	Kovach *et al*. (1994)
pBBR1MCS-5	Broad-host-range vector, IncP, -W, -Q, ColE1 and p15A compatible, contains pBluescript IIKS-*lacZα*-polylinker, Gm^r^	Kovach *et al*. (1995)
pSRKKan	pBBR1MCS derivative, broad-host-range inducible expression vector, Km^r^	Khan *et al*. (2008)
pSRKGm	pBBR1MCS derivative, broad-host-range inducible expression vector, Gm^r^	Khan *et al*. (2008)
pET28a_*flaA*	pET28 derivative used to express His-tagged fusion of FlaA in *E. coli* BL21(DE3), Km^r^	Parker *et al*. (2012)
pET28a_*flaA-CBD*	pET28 derivative used to express His-tagged fusion of FlaA-CBD in *E. coli* BL21(DE3), Km^r^	This work
pSRK_*hisflaA*	pSRK derivative used to express His-tagged fusion of FlaA in *A. caviae*, Gm^r^	Parker *et al*. (2012)
pSRK_*flaA*	pSRK derivative used to express *flaA* in *A. caviae*, Gm^r^	This work
pSRK_*flaA-CBD*	pSRK derivative used to express *flaA*-CBD in *A. caviae*, Gm^r^	This work
pBBR1MCS_*maf1*	pBBR1MCS with *A. caviae maf1* and 170 bp upstream region, Cm^r^	Parker *et al*. (2012)
pBBR1MCS_*maf1HIS*	pBBR1MCS with *A. caviae maf1* and 170 bp upstream region, Cm^r^ with C-terminal 5xHistag	This work
pET28_*flaJ*	pET28 derivative used to express His-tagged fusion of FlaJ in *E. coli* BL21(DE3), Km^r^	This work
pGEX4T3_*flaJ*	pGEX4T3 derivative used to express GST-tagged fusion of FlaJ in *E. coli* BL21(DE3), Amp^r^	This work

### Motility assays

To assess motility of *Aeromonas* strains, bacterial colonies were transferred with a sterile toothpick into the centre of plates containing motility agar (1% tryptone, 0.5% NaCl, 0.3% agar). The plates were incubated face up at 37°C for 14–24 h, and motility was assessed by examining the migration of bacteria through the agar from the centre towards the periphery of the plate.

### Secretion assays

Overnight cultures of *A. caviae* strains were used to inoculate 10 ml of LB (1:100). Cultures were grown at 37°C with shaking to late-log phase whole-cell preparations carried out as previously described (Tabei *et al*., 2009; Parker *et al*., 2012). Secreted fractions were generated by precipitating culture supernatant with two volumes of ice-cold ethanol. Samples were centrifuged at 50 000 *g* for 20 min and protein pellets were washed in acetone, dried and resuspended in 200 μl PBS. Samples were analysed by SDS-PAGE and Western blotting (as described below).

### Isolation of flagellin from *A. caviae*

For isolation of glycosylated flagellin filaments from *A. caviae*, TSB agar grown cultures were harvested in PBS and subjected to shearing forces using a laboratory blender (Wilhelms *et al*., 2012). Cells were removed by centrifugation at 8000 *g*, the supernatant was subjected to an additional centrifugation step at 18 000 *g* to remove further cell debris and agar. The flagellar filaments were then pelleted during a final centrifugation step of 2 h at 75 000 *g*. Flagellin pellets were resuspended in an appropriate volume of PBS. For isolation of flagellin from a *maf1* mutant strain of *A. caviae*, culture supernatant was precipitated with two volumes of ice-cold ethanol and underwent separation by SDS-PAGE.

### Protein purification methods

For purification of GST-tagged proteins from *E. coli* and His-tagged proteins from *A. caviae* (Maf1), and *E. coli* (FlaA, FlaA-CBD, FlaJ), *A. caviae flaJ* was cloned into the protein expression vector pGEX4T3 (BamHI) allowing expression of N-terminal GST-tagged fusion proteins, and *A. caviae flaA, flaA-CBD* and *flaJ* was cloned into the protein expression vector pET28a (NdeI, BamHI) allowing expression N-terminal His-tagged fusion proteins. For expression in *A. caviae*, the pBBR1MCS_*maf1* plasmid was amplified using overlapping primers, introducing a C-terminal penta-his. Recombinant *E. coli* BL21(DE3) harbouring pGEX4T3_*flaJ*, pET28a_*flaA,* pET28a_*flaA-CBD,* pET28a_*flaJ,* and *A. caviae* strains harbouring pBBR1MCS_*maf1his* were used to inoculate 10 ml of LB supplemented with appropriate antibiotics and incubated at 37°C with shaking overnight. The 10 ml overnight grown culture was used to inoculate 1 litre of LB (1:1000) supplemented with appropriate antibiotics and incubated at 37°C with shaking. For BL21(DE3) IPTG was added to a final concentration of 1 mM when cell density reached OD_600_ = 0.5 and growth was continued for an additional 2 h. Growth of *A. caviae* strains was carried out for 8 h. Cells were harvested by centrifugation. For glutathione-affinity chromatography the cell pellet was resuspended in GST lysis buffer [50 mM Tris-HCl (pH 8), 150 mM NaCl, 10% glycerol, 1 mM EDTA, 0.1 mg ml^−1^ lysozyme] supplemented with complete protease inhibitor tablets, DNase, and RNase (Roche) according to the manufacturer's instructions. Cells were disrupted by sonication. The lysate was centrifuged at 27 000 *g* for 60 min at 4°C. The supernatant was incubated with 0.5 ml glutathione sepharose 4B resin (GE Healthcare) for 2 h with end over end rotation. Resin was extensively washed with GST wash buffer [50 mM Tris-HCl (pH 8), 150 mM NaCl, 10% glycerol] and GST-tagged proteins were eluted from the resin with elution buffer [50 mM Tris-HCl (pH 8), 15 mM glutathione]. For nickel-affinity chromatography the cell pellet was resuspended in lysis buffer [20 mM phosphate (pH 7.4), 50 mM imidazol, 0.5 M NaCl, 0.1 mg ml^−1^ lysozyme] supplemented with complete protease inhibitor tablets, DNase, and Rnase (Roche). Cells were disrupted by sonication and the lysate centrifuged as above. For soluble proteins (HisFlaJ and Maf1His) the supernatant was collected and applied to a column packed with an appropriate volume of Ni-NTA superflow resin (Qiagen, UK) that had been equilibrated with *E. coli* lysis buffer The resin was washed extensively with wash buffer [20 mM phosphate buffer (pH 7.4), 0.5 M NaCl, 50 mM imidazole] and eluted with a stepwise gradient of imidazol (50–500 mM). For insoluble proteins the insoluble pellet was resuspended in insoluble wash buffer [20 mM phosphate buffer (pH 7.4), 0.5 M NaCl, 50 mM imidazole, 8 M urea] and centrifuged at 27 000 *g* for 30 min at 4°C. The supernatant was collected and treated the same way, with the addition of 8 M urea in all of the buffers. The presence of the protein of interest was detected by SDS-PAGE and Western blotting. Samples were dialysed against PBS.

### SDS-PAGE and immunoblotting

Cell fractions from *A. caviae* were obtained from BHIB cultures grown to mid-log phase at 37°C. Equivalent numbers of cells were harvested by centrifugation and harvested cell pellets were resuspended in SDS-PAGE sample loading buffer and mixed 1:1 with PBS. Samples were boiled and separated by SDS-PAGE (12% acrylamide). Protein samples that had undergone nickel-affinity chromatography or glutathione-affinity chromatography were mixed 1:1 with SDS-PAGE sample loading buffer and analysed the same way. Western blot analyses of protein samples were performed as previously described using the following primary antibodies; alpha-rabbit FlaA/Bpse (1:20 000); alpha-rat FlaA/B (1:10 000); alpha-rabbit GroEL (1:100 000 Sigma); alpha-mouse Penta-His (1:5000 Qiagen). Rabbit (Qiagen), rat (Cell signalling technology), and mouse (Qiagen) HRP-conjugated secondary antibodies were used for detection (1:5000). Analysis of GST-tagged proteins used αGST-HRP-conjugate (GE Healthcare) (1:5000). The bound conjugate was then detected using the ECL detection system (GE Healthcare). For far Western analysis membranes were incubated in PBS with purified GST or GST-FlaJ at 0.01 mg ml^−1^. After gentle washing, the membrane was incubated with αGST-HRP-conjugate (GE Healthcare) (1:5000). The bound conjugate was then detected using the ECL detection system (GE Healthcare). For detection of the ECL signal, the membrane was exposed to X-ray film, adjusting the exposure time to allow for optimization of the signal. Resulting bands were subjected to densitometry for binding analyses.

### Trypsin digest of flagellin

In-gel trypsin digestion was performed as previously described (Shevchenko *et al*., 2006; Couto *et al*., 2011). Gel bands of interest were excised from a polyacrylamide gel after SDS-PAGE and transferred to clean LoBind Eppendorf tubes. Gel pieces were de-stained with 100 mM ammonium bicarbonate/acetonitrile solution (50:50, v/v) and dehydrated with 100% acetonitrile. Reduction and alkylation was carried out with 10 mM of dl-dithiothreitol (DTT) and 55 mM iodoacetamide respectively. In-gel digestion with trypsin (Promega) was performed at a ratio of 20:1 (protein : enzyme; w/w) (0.5 μg) in 100 mM ammonium bicarbonate/5% (v/v) formic acid at 37°C for 16 h. After digestion, peptides were extracted and dried in a vacuum centrifuge and stored at −20°C.

### LC-MS/MS analysis of flagellins

A U3000 nanoflow HPLC system (ThermoFisher, Hemel Hempstead, UK) was directly connected to the mass spectrometer [maXis^TM^ UHR-Qq-ToF (Bruker Daltonic, Bremen, Germany)], fitted with an EZ Nanoflow Electrospray needle. The U3000 HPLC system was equipped with a nanoLC analytical column [75 μm × 15 cm packed with C18 material, 5 μm, 100 Å particles (LC Packings, CA, USA)] and micro precolumn [300 μm i.d. × 5 packed with C18 material, 5 μm, 100 Å particles (LC Packings, CA, USA)] at flow rates of 300 nl min^−1^ and 30 μl min^−1^ respectively. A linear HPLC gradient using buffer A [97% (v/v) HPLC water, 3% (v/v) HPLC acetonitrile with 0.1% (v/v) formic acid] and buffer B [97% (v/v) HPLC acetonitrile, 3% (v/v) HPLC grade water with 0.1% (v/v) formic acid], using a 120 min programme, was applied as follows: 0% B (0–5 min), 0–35% B (5–95 min), 35–100% B (95–101 min), 100% B (101–106 min), 0% B (106–120 min). Acquired data were analysed initially using EasyProt (Gluck *et al*., 2013) and then further analysed manually using the mass of precursor/fragment ions predicted from *in silico* digests using the protein product tool on the Protein Prospector website (http://prospector.ucsf.edu/prospector/mshome.htm).

## References

[b1] Akeda Y, Galan JE (2005). Chaperone release and unfolding of substrates in type III secretion. Nature.

[b2] Arnold R, Brandmaier S, Kleine F, Tischler P, Heinz E, Behrens S (2009). Sequence-based prediction of type III secreted proteins. PLoS Pathog.

[b3] Auvray F, Thomas J, Fraser GM, Hughes C (2001). Flagellin polymerisation control by a cytosolic export chaperone. J Mol Biol.

[b4] Bennett JC, Hughes C (2000). From flagellum assembly to virulence: the extended family of type III export chaperones. Trends Microbiol.

[b5] Blocker A, Jouihri N, Larquet E, Gounon P, Ebel F, Parsot C (2001). Structure and composition of the *Shigella flexneri* ‘needle complex’, a part of its type III secreton. Mol Microbiol.

[b6] Canals R, Vilches S, Wilhelms M, Shaw JG, Merino S, Tomas JM (2007). Non-structural flagella genes affecting both polar and lateral flagella-mediated motility in *Aeromonas hydrophila*. Microbiology.

[b7] Couto N, Barber J, Gaskell SJ (2011). Matrix-assisted laser desorption/ionisation mass spectrometric response factors of peptides generated using different proteolytic enzymes. J Mass Spectrom.

[b8] Eaton KA, Suerbaum S, Josenhans C, Krakowka S (1996). Colonization of gnotobiotic piglets by *Helicobacter pylori* deficient in two flagellin genes. Infect Immun.

[b9] Erhardt M, Namba K, Hughes KT (2010). Bacterial nanomachines: the flagellum and type III injectisome. Cold Spring Harb Perspect Biol.

[b10] Evans LD, Stafford GP, Ahmed S, Fraser GM, Hughes C (2006). An escort mechanism for cycling of export chaperones during flagellum assembly. Proc Natl Acad Sci USA.

[b11] Evdokimov AG, Phan J, Tropea JE, Routzahn KM, Peters HK, Pokross M, Waugh DS (2003). Similar modes of polypeptide recognition by export chaperones in flagellar biosynthesis and type III secretion. Nat Struct Biol.

[b12] Ewing CP, Andreishcheva E, Guerry P (2009). Functional characterization of flagellin glycosylation in *Campylobacter jejuni* 81-176. J Bacteriol.

[b13] Gluck F, Hoogland C, Antinori P, Robin X, Nikitin F, Zufferey A (2013). EasyProt – an easy-to-use graphical platform for proteomics data analysis. J Proteomics.

[b14] Goon S, Kelly JF, Logan SM, Ewing CP, Guerry P (2003). Pseudaminic acid, the major modification on *Campylobacter* flagellin, is synthesized via the *Cj1293* gene. Mol Microbiol.

[b15] Gryllos I, Shaw JG, Gavin R, Merino S, Tomas JM (2001). Role of *flm* locus in mesophilic *Aeromonas* species adherence. Infect Immun.

[b16] Guerry P, Ewing CP, Schirm M, Lorenzo M, Kelly J, Pattarini D (2006). Changes in flagellin glycosylation affect *Campylobacter* autoagglutination and virulence. Mol Microbiol.

[b17] Hayashi F, Smith KD, Ozinsky A, Hawn TR, Yi EC, Goodlett DR (2001). The innate immune response to bacterial flagellin is mediated by Toll-like receptor 5. Nature.

[b18] Howard SL, Jagannathan A, Soo EC, Hui JP, Aubry AJ, Ahmed I (2009). *Campylobacter jejuni* glycosylation island important in cell charge, legionaminic acid biosynthesis, and colonization of chickens. Infect Immun.

[b19] Iwashkiw JA, Vozza NF, Kinsella RL, Feldman MF (2013). Pour some sugar on it: the expanding world of bacterial protein O-linked glycosylation. Mol Microbiol.

[b20] Josenhans C, Vossebein L, Friedrich S, Suerbaum S (2002). The *neuA**flmD* gene cluster of *Helicobacter pylori* is involved in flagellar biosynthesis and flagellin glycosylation. FEMS Microbiol Lett.

[b21] Jyot J, Gautam JK, Raje M, Ghosh A (1999). Localization of DnaK and GroEL in *Vibrio cholerae*. FEMS Microbiol Lett.

[b22] Karlyshev AV, Linton D, Gregson NA, Wren BW (2002). A novel paralogous gene family involved in phase-variable flagella-mediated motility in *Campylobacter jejuni*. Microbiology.

[b23] Khan SR, Gaines J, Roop RM, Farrand SK (2008). Broad-host-range expression vectors with tightly regulated promoters and their use to examine the influence of TraR and TraM expression on Ti plasmid quorum sensing. Appl Environ Microbiol.

[b24] Kirov SM, Castrisios M, Shaw JG (2004). *Aeromonas* flagella (polar and lateral) are enterocyte adhesins that contribute to biofilm formation on surfaces. Infect Immun.

[b25] Kovach ME, Phillips RW, Elzer PH, Roop RM, Peterson KM (1994). pBBR1MCS: a broad-host-range cloning vector. Biotechniques.

[b26] Kovach ME, Elzer PH, Hill DS, Robertson GT, Farris MA, Roop RM, Peterson KM (1995). Four new derivatives of the broad-host-range cloning vector pBBR1MCS, carrying different antibiotic-resistance cassettes. Gene.

[b27] Lertsethtakarn P, Ottemann KM, Hendrixson DR (2011). Motility and chemotaxis in *Campylobacter* and *Helicobacter*. Annu Rev Microbiol.

[b28] Logan SM (2006). Flagellar glycosylation – a new component of the motility repertoire?. Microbiology.

[b29] de Lorenzo V, Herrero M, Jakubzik U, Timmis KN (1990). Mini-Tn5 transposon derivatives for insertion mutagenesis, promoter probing, and chromosomal insertion of cloned DNA in gram-negative eubacteria. J Bacteriol.

[b30] McNally DJ, Hui JP, Aubry AJ, Mui KK, Guerry P, Brisson JR (2006). Functional characterization of the flagellar glycosylation locus in *Campylobacter jejuni* 81-176 using a focused metabolomics approach. J Biol Chem.

[b31] Merino S, Rubires X, Aguilar A, Tomas JM (1997). The role of flagella and motility in the adherence and invasion to fish cell lines by *Aeromonas hydrophila* serogroup O:34 strains. FEMS Microbiol Lett.

[b32] Muskotal A, Kiraly R, Sebestyen A, Gugolya Z, Vegh BM, Vonderviszt F (2006). Interaction of FliS flagellar chaperone with flagellin. FEBS Lett.

[b33] Nothaft H, Szymanski CM (2010). Protein glycosylation in bacteria: sweeter than ever. Nat Rev Microbiol.

[b34] Parker JL, Day-Williams MJ, Tomas JM, Stafford GP, Shaw JG (2012). Identification of a putative glycosyltransferase responsible for the transfer of pseudaminic acid onto the polar flagellin of *Aeromonas caviae* Sch3N. Microbiologyopen.

[b35] Paul K, Erhardt M, Hirano T, Blair DF, Hughes KT (2008). Energy source of flagellar type III secretion. Nature.

[b36] Pratt LA, Kolter R (1998). Genetic analysis of *Escherichia coli* biofilm formation: roles of flagella, motility, chemotaxis and type I pili. Mol Microbiol.

[b37] Rabaan AA, Gryllos I, Tomas JM, Shaw JG (2001). Motility and the polar flagellum are required for *Aeromonas caviae* adherence to HEp-2 cells. Infect Immun.

[b38] Schirm M, Soo EC, Aubry AJ, Austin J, Thibault P, Logan SM (2003). Structural, genetic and functional characterization of the flagellin glycosylation process in *Helicobacter pylori*. Mol Microbiol.

[b39] Schirm M, Kalmokoff M, Aubry A, Thibault P, Sandoz M, Logan SM (2004). Flagellin from *Listeria monocytogenes* is glycosylated with beta-O-linked N-acetylglucosamine. J Bacteriol.

[b40] Schoenhofen IC, McNally DJ, Brisson JR, Logan SM (2006). Elucidation of the CMP-pseudaminic acid pathway in *Helicobacter pylori*: synthesis from UDP-N-acetylglucosamine by a single enzymatic reaction. Glycobiology.

[b41] Schoenhofen IC, Vinogradov E, Whitfield DM, Brisson JR, Logan SM (2009). The CMP-legionaminic acid pathway in *Campylobacter*: biosynthesis involving novel GDP-linked precursors. Glycobiology.

[b42] Shevchenko A, Tomas H, Havlis J, Olsen JV, Mann M (2006). In-gel digestion for mass spectrometric characterization of proteins and proteomes. Nat Protoc.

[b43] Tabei SM, Hitchen PG, Day-Williams MJ, Merino S, Vart R, Pang PC (2009). An *Aeromonas caviae* genomic island is required for both O-antigen lipopolysaccharide biosynthesis and flagellin glycosylation. J Bacteriol.

[b44] Tapia-Pastrana G, Chavez-Duenas L, Lanz-Mendoza H, Teter K, Navarro-Garcia F (2012). VirK is a periplasmic protein required for efficient secretion of plasmid-encoded toxin from enteroaggregative *Escherichia coli*. Infect Immun.

[b45] Thibault P, Logan SM, Kelly JF, Brisson JR, Ewing CP, Trust TJ, Guerry P (2001). Identification of the carbohydrate moieties and glycosylation motifs in *Campylobacter jejuni* flagellin. J Biol Chem.

[b46] Twine SM, Paul CJ, Vinogradov E, McNally DJ, Brisson JR, Mullen JA (2008). Flagellar glycosylation in *Clostridium botulinum*. FEBS J.

[b47] Verma A, Arora SK, Kuravi SK, Ramphal R (2005). Roles of specific amino acids in the N terminus of *Pseudomonas aeruginosa* flagellin and of flagellin glycosylation in the innate immune response. Infect Immun.

[b48] Verma A, Schirm M, Arora SK, Thibault P, Logan SM, Ramphal R (2006). Glycosylation of b-Type flagellin of *Pseudomonas aeruginosa*: structural and genetic basis. J Bacteriol.

[b49] Wilhelms M, Fulton KM, Twine SM, Tomas JM, Merino S (2012). Differential glycosylation of polar and lateral flagellins in *Aeromonas hydrophila* AH-3. J Biol Chem.

[b50] Yonekura K, Maki S, Morgan DG, DeRosier DJ, Vonderviszt F, Imada K, Namba K (2000). The bacterial flagellar cap as the rotary promoter of flagellin self-assembly. Science.

[b51] Yonekura K, Maki-Yonekura S, Namba K (2003). Complete atomic model of the bacterial flagellar filament by electron cryomicroscopy. Nature.

