# Supplementary material for: Maf-dependent bacterial flagellin glycosylation occurs before chaperone binding and flagellar T3SS export
Source: Mol Microbiol. 2014 Mar 4;92(2):258–72. doi: 10.1111/mmi.12549 (PMC4065374; doi:10.1111/mmi.12549)
Supplement: Supplementary file 1 [file mmi0092-0258-SD1.pdf]

Supplementary Table 1: Oligonucleotides used in this study

Primer name	Sequence 5' to 3' (Restriction site)	Gene/Use
JLP_01F	ATATATATCATATGTACAAGCGCAATATC (NdeI)	Amplification of *flaJ* to generate pET28_*flaJ*
JLP_02R	TATTATGGATCCTCATAGCTCACCCAACTG (BamHI)	Amplification of *flaJ* to generate pET28_*flaJ*
JLP_05F	ATATATGGATCCATGTACAAGCGCAATATCAAAG (BamHI)	Amplification of *flaJ* to generate pGEX4T3_*flaJ* (used with JLP_02)
JLP_20F	ATATATCATATGATGAGTCTGTATATC (NdeI)	Amplification of *flaA* to generate pET28_*flaA and* pSRK_*flaA*
JLP_21R	TATTATGGATCCTTAGTTCTGCAGCAG (BamHI)	Amplification of *flaA* to generate pET28_*flaA and* pSRK_*flaA*
JLP_90R	TATGGATCCTTATGCATCGCGGATACGAG (BamHI)	Amplification of *flaA-cbd* to generate pET28_*flaA-cbd* and pSRK_*flaA-cbd* (used with JLP_20)
JLP_166F	CGATAGTGTCGATATGATAGACAATGAAGTTATTGTACTATTCAAAAAACATCATCACCATCATCATTAAGCGGCCGCCACCGCGGTGGAGC	Amplification of pBBR1MCS_*maf1*using QuikChange® primers (Agilent technologies) to introduce a C-terminal 5xHis-tag
JLP_167R	GGAGCTCCACCGCGGTGGCGGCCGCTTAATGATGATGGTGATGATGTTTTTTGAATAGTACAATAACTTCATTGTCTATCATATCGACACTATCG	Amplification of pBBR1MCS_*maf1*using QuikChange® primers (Agilent technologies) to introduce a C-terminal 5xHis-tag
